# Supplementary material for: Comprehensive analysis reveals potential therapeutic targets and an integrated risk stratification model for solitary fibrous tumors
Source: Nat Commun. 2023 Nov 18;14:7479. doi: 10.1038/s41467-023-43249-4 (PMC10657378; doi:10.1038/s41467-023-43249-4)
Supplement: Supplementary file 1 — Supplementary Information [file 41467_2023_43249_MOESM1_ESM.pdf]

Supplementary Information

Comprehensive analysis reveals potential therapeutic targets and an integrated risk stratification model for solitary fibrous tumors

Renjing Zhang[1,2#], Yang Yang[3#], Chunfang Hu[4#], Mayan Huang[1,5#], Wenjian Cen[1,2#], Dongyi Ling[1,2], Yakang Long[1,2], Xin-Hua Yang[1,2], Boheng Xu[1,2], Junling Peng[1,2], Sujie Wang[1,2], Weijie Zhu[1,2], Mingbiao Wei[1,2], Jiaojiao Yang[1,2], Yuxia Xu[1,2], Xu Zhang[1,2], Jiangjun Ma[1,2], Fang Wang[1,2], Hongtu Zhang[4], Peiqing Ma[4], Xiaojun Zhu[1,6], Guohui Song[1,6], Li-Yue Sun[7], De-Shen Wang[1,8], Feng-Hua Wang[1,8], Yu-Hong Li[1,8], Sandro Santagata[9], Qin Li[10,11*], Yan-Fen Feng[1,5*], Ziming Du[1,2*]

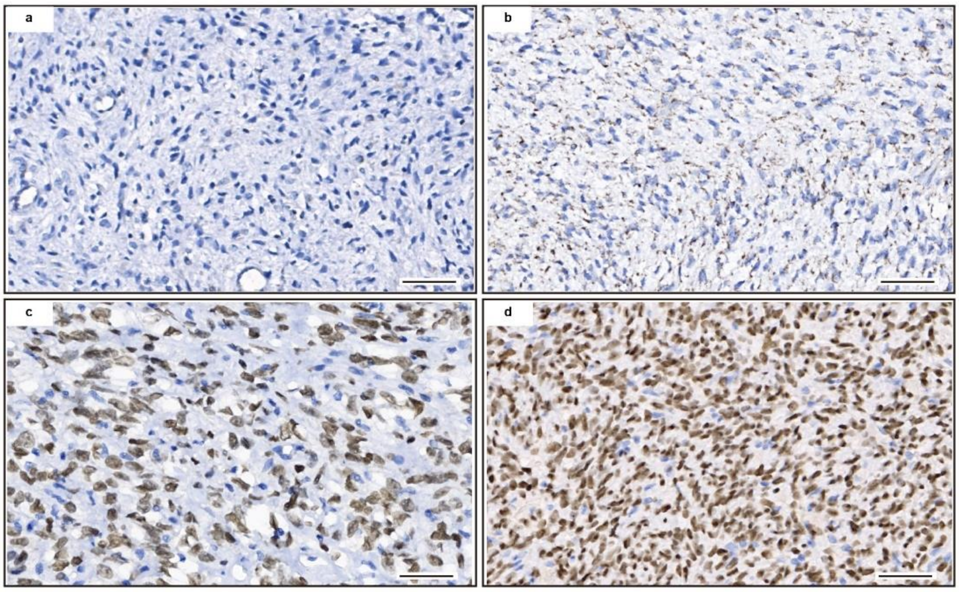

Supplementary Fig. 1: STAT6 staining in solitary fibrous tumors. Nuclear and cytoplasmic staining was qualitatively scored as 0 (absent to minimal blush or staining of <10% of cells, any intensity), or 1+ (present in 10% or more of cells, weak physiologic stain equivalent to intensity seen in intratumoral lymphocytes), or 2+ (moderate overexpression), or 3+ (high-level overexpression). **a,** Representative image of score 0, both nuclear and cytoplasmic are absent of stain. **b,** Score 1+, cytoplasmic stain. **c,** Score 2+, moderate nuclear stain. **d,** Score 3+，strong nuclear stain. (Scale bar = 50 μm)

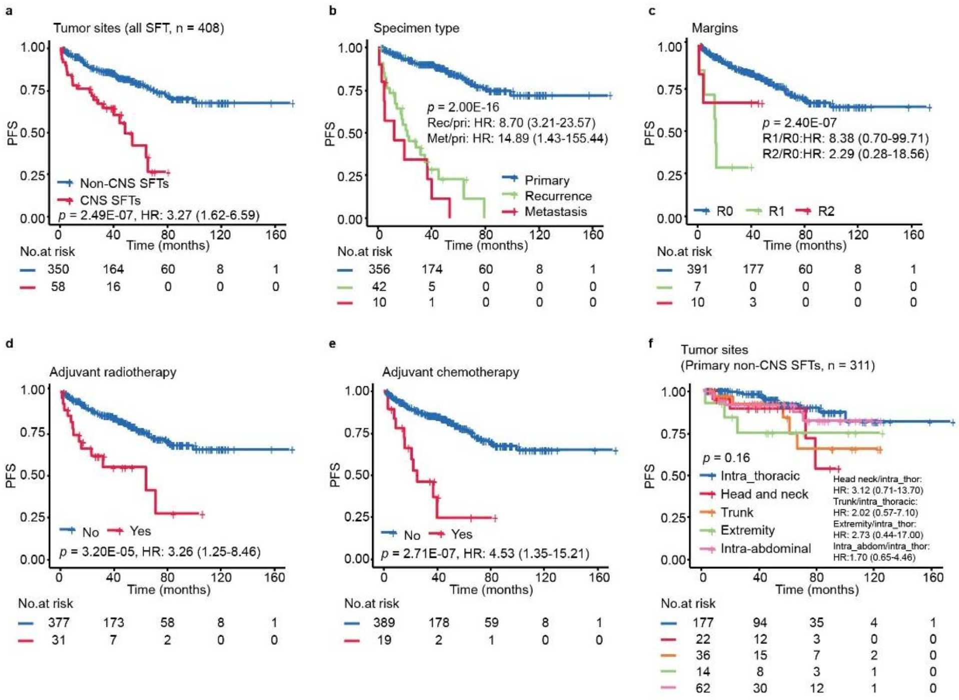

Supplementary Fig. 2: Prognostic value of clinical variables in all 408 SFTs from four cohorts. a, Kaplan-Meier plots showing PFS for CNS SFTs and non-CNS SFTs. **b,** Kaplan-Meier plots showing PFS for primary SFTs, recurrent SFTs and metastatic SFTs. **c**, Kaplan-Meier plots showing PFS for the SFTs with tumor margin of R0, R1 and R2. **d,** Kaplan-Meier plots showing PFS for the SFT patients with or without adjuvant radiotherapy. **e,** Kaplan-Meier plots showing PFS for the SFT patients with or without adjuvant chemotherapy. **f,** Kaplan-Meier plots showing PFS for the non-CNS SFTs originated from different sites. *p* values were calculated using two-sided log-rank test. SFT Solitary Fibrous Tumor; CNS Central Nervous System; HR Hazard Ratio; R0 Microscopic Complete; R1 Microscopic Incomplete; R2 Macroscopic Incomplete; PFS Progression Free Survival.

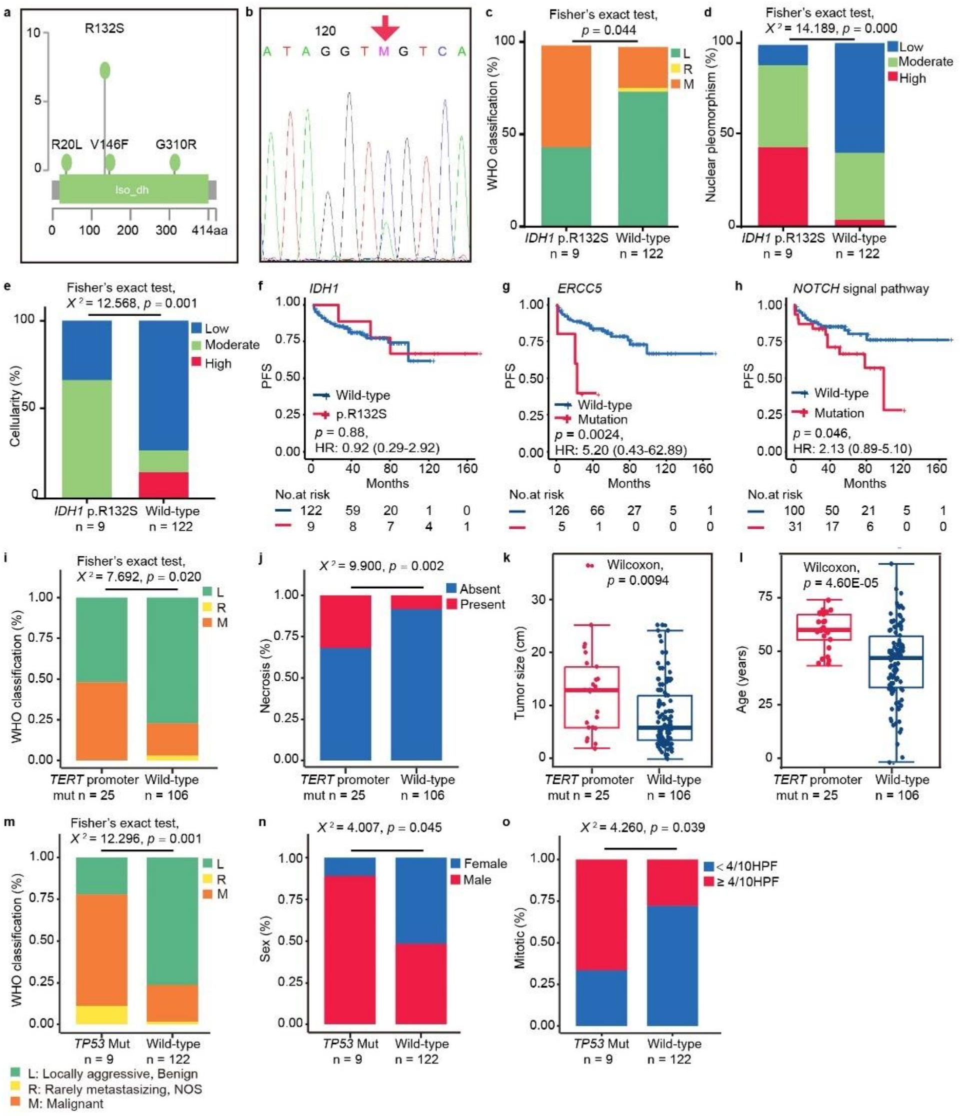

Supplementary Fig. 3: The association between gene mutations and clinicopathologic parameters. a, Mutant lollipop indicated the hotspot mutation site of *IDH1* p.R132S (n = 7) in SFTs. **b**, Sanger sequencing confirmed *IDH1* p.R132S mutation in 7 SFTs in SYSUCC cohort. **c-f,** *IDH1* p.R132S mutation was associated with WHO classification, nuclear pleomorphism and cellularity but not associated with patients PFS. *p* values were calculated using two-sided log-rank test (f). **g-h**, Kaplan-Meier plots showing PFS for patients who had mutation and wild type of *ERCC5*, *NOTCH* signal pathway, *p* values were calculated using two-sided log-rank test. **i-l,** *TERT* promoter region mutations were enriched in malignant tumor in WHO classification, tumors with necrosis, and positively correlated with large size of tumors (tumor size: 13.00

(IQR: 6.00-17.65) cm vs 6.05 (IQR: 3.75-12.13) cm) and age of the patients (age: 61.00 (IQR: 55.50-67.00) years vs 50.00 (IQR: 38.00-59.25) years). Box center lines, bounds of the box, and whiskers indicate medians, first and third quartiles, and minimum and maximum values within 1.5×IQR (interquartile range) of the box limits, respectively. *p* values were calculated using Wilcoxon test (k, l). **m-o,** *TP53* mutations were enriched in malignant tumors in WHO classification, male patients, and high mitotic tumors. WHO World Health Organization; PFS Progression Free Survival; HR Hazard Ratio.

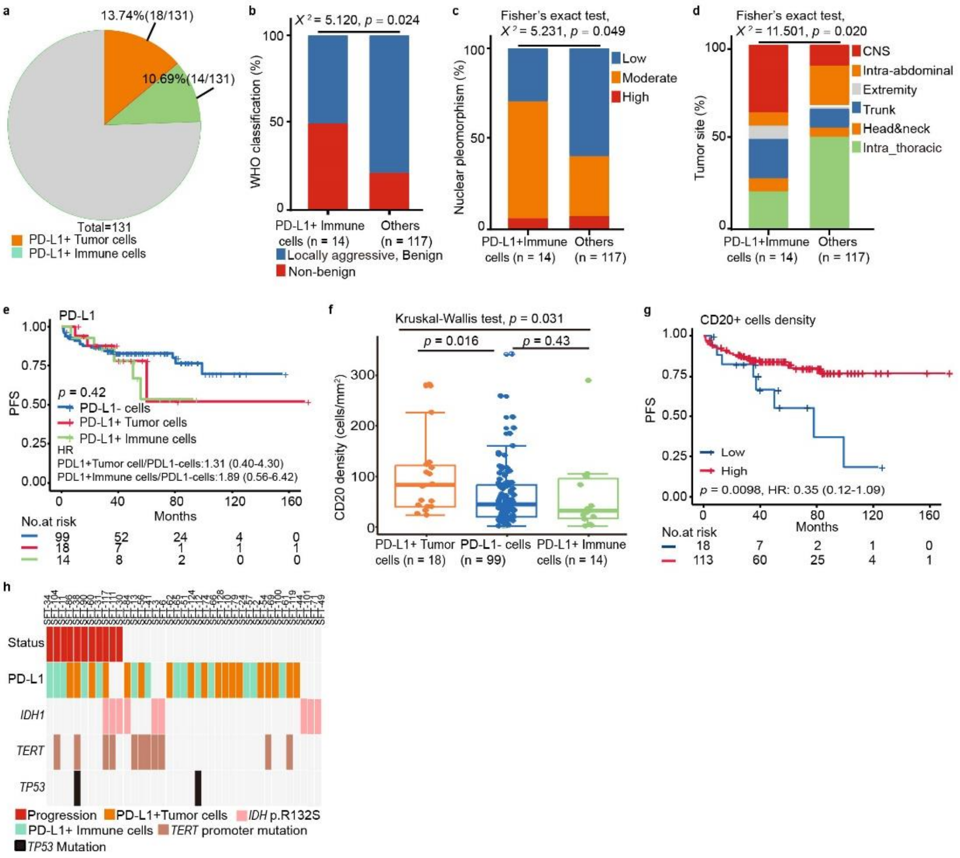

Supplementary Fig. 4: High density of macrophages and high PD-L1 expression were found in SFTs. a, PD-L1 was highly expressed in 13.74% (18/131) of SFT tissues in tumor cells, and 10.69% (14/131) of SFT tissues in immune cells (mostly macrophages). **b-d**, High immune cells expressing PD-L1 was enriched in malignant or rarely metastasizing samples in WHO classification, high nuclear pleomorphism, and in SFTs originated from CNS. **e,** Kaplan-Meier plots showing PFS for patients with PD-L1+ tumor cells, patients with PD-L1+ immune cells, and patients with PD-L1- cells, *p* values were calculated using two-sided log-rank test. **f,** High density of CD20+ B cells was enriched in SFTs with tumor cells expressing PD-L1 (the median density of B cells in PD-L1 + tumor cell, others and PD-L1+ immune cells tissues were 86.25 (IQR: 42.10-129.18), 47.70 (IQR: 22.30-87.60), 34.55 (IQR: 13.98-103.98) cells/mm^2). Box center lines, bounds of the box, and whiskers indicate medians, first and third quartiles, and minimum and maximum values within 1.5×IQR

(interquartile range) of the box limits, respectively. *p* values were calculated using Kruskal-Wallis test. **g,** Kaplan-Meier plots showing PFS for patients with high or low density of CD20+ B cells infiltrated in SFTs tissues. *p* values were calculated using two-sided log-rank test. **h,** Oncoprint plot showing the distribution of high PD-L1 expression and gene alteration of *IDH1*, *TERT*, and *TP53* in SFTs from SYSUCC cohort. WHO World Health Organization; CNS Central Nervous System; PFS Progression Free Survival; HR Hazard Ratio.

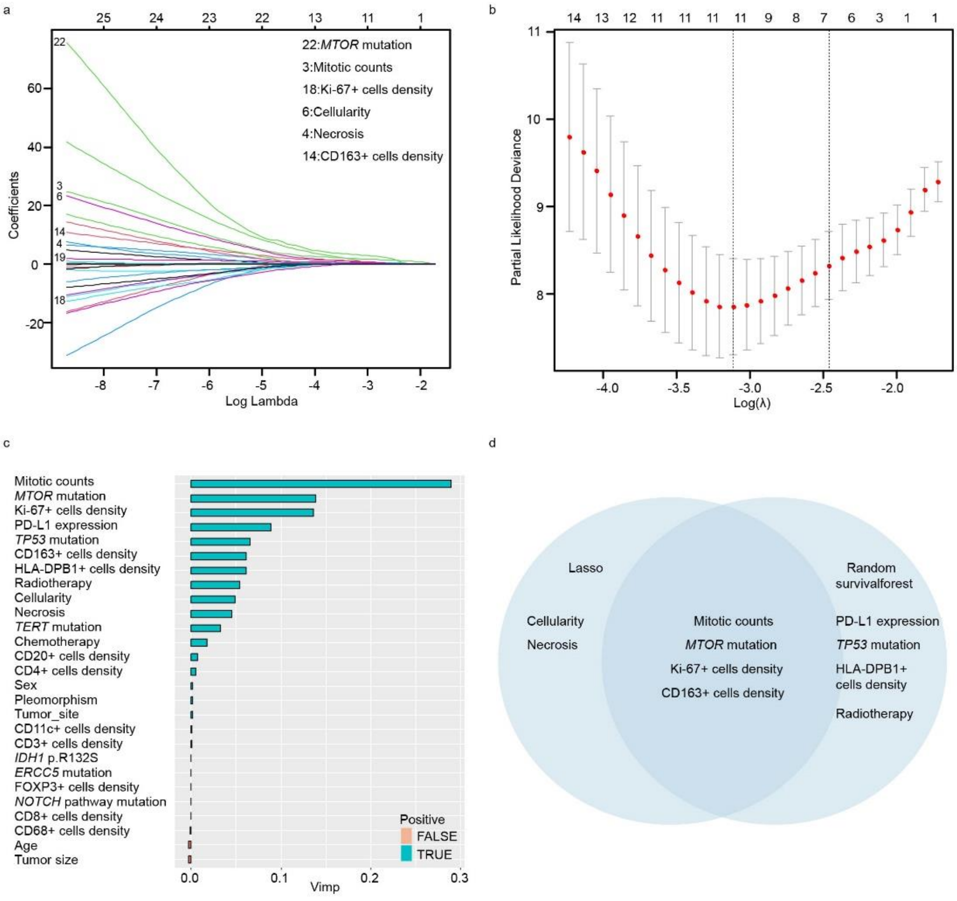

Supplementary Fig. 5: Evaluation of variables for integrated risk model. a, LASSO regression was used to screen and rank variables which initially included clinical and histopathological factors (including mitotic count, necrosis, age, sex, tumor size, tumor site, cellularity and nuclear pleomorphism), immunohistochemical factors (including the density of cells which are Ki-67+, CD68+, CD163+, HLA-DPB1+, PD-L1+, CD3+, CD4+, CD8+, FOXP3+, CD11c+, CD20, receptively) and molecular factors (including *MTOR* mutation, *NOTCH* pathway mutation (*NOTCH1*, *NOTCH2*, *NOTCH3*, *CREBBP*), *ERCC5* mutation, *TP53* mutation, *TERT* promoter region mutation and *IDH1* mutation). **b,** LASSO regression cross-validation, fitting, and selection of models. **c,** the variables screened and ranked by random survival forest VIMP according to their importance. **d,** The common variables selected by both LASSO regression and random survival forest.

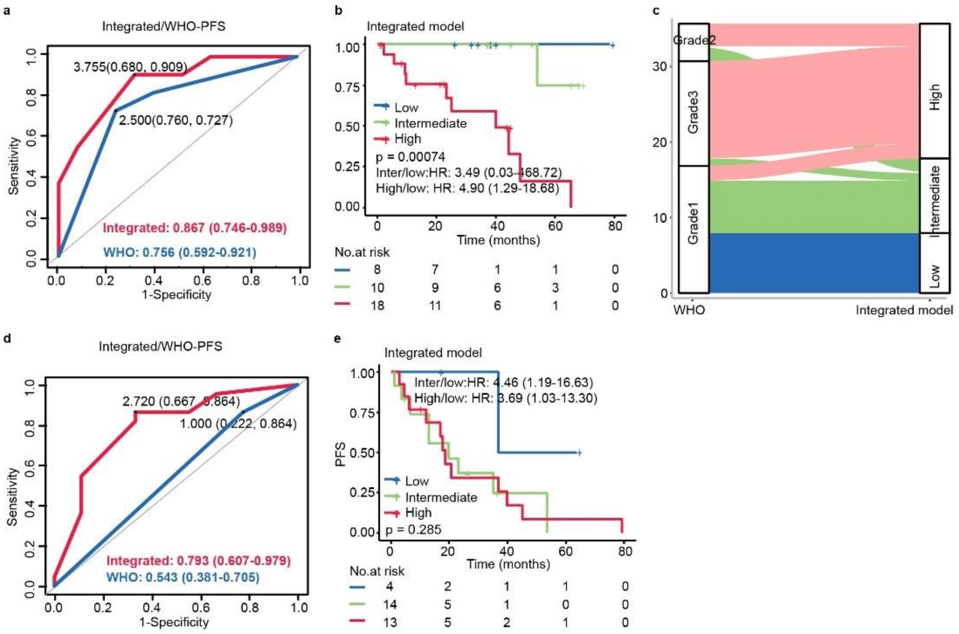

Supplementary Fig. 6: The performance of integrated risk model in primary CNS SFTs with NTM and relapsed non-CNS SFTs with NTM. a, ROC curves for integrated risk model, WHO grade in primary CNS SFTs with NTM from four cohorts. **b**, Kaplan-Meier plots showing PFS for primary CNS SFTs with NTM stratified by integrated risk model, *p* values were calculated using two-sided log-rank test. **c**, Sankey diagram showing the intersection of the SFT patients stratified by both WHO classification and integrated risk model. **d**, ROC curves for integrated risk model, WHO classification in relapsed non-CNS SFTs with NTM from the four cohorts. **e**, Kaplan-Meier plots showing PFS for relapsed non-CNS SFTs with NTM stratified by integrated risk model, *p* values were calculated using two-sided log-rank test. PFS Progression Free Survival; HR Hazard Ratio; WHO World Health Organization; Source data are provided as a Source Data file.

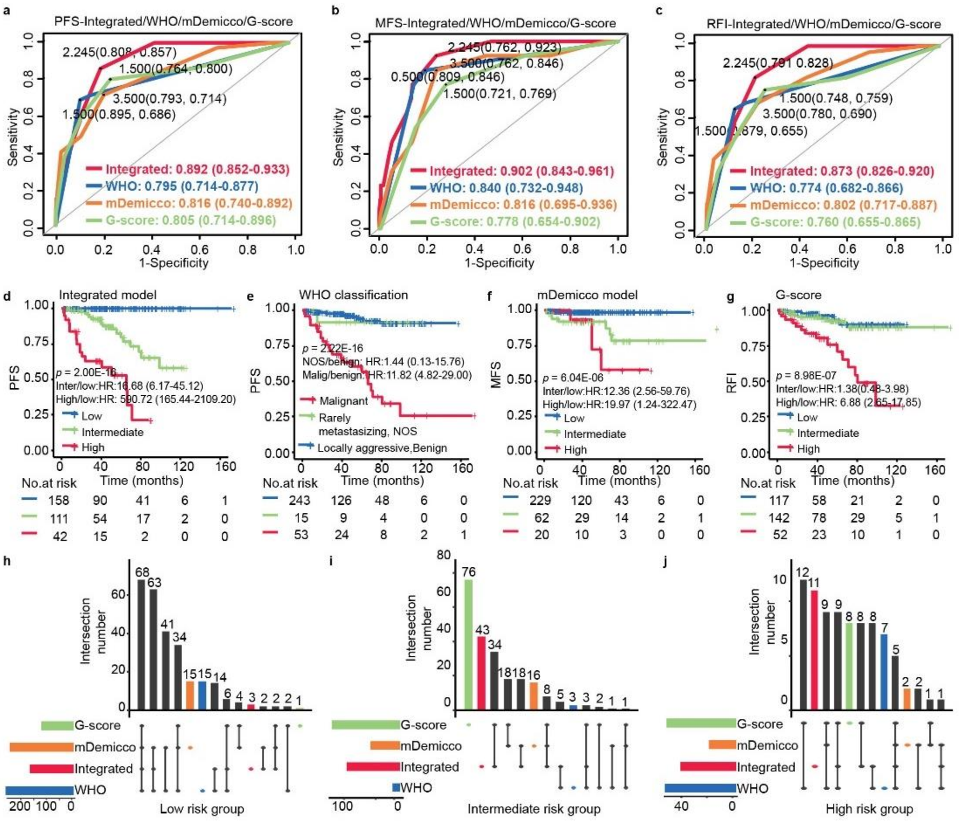

Supplementary Fig. 7: Comparison of the integrated risk model to WHO classification and published models under the stricter criteria. All primary non-CNS SFTs with NTM from four cohorts were included (n = 311), and in addition to PFS, MFS and RFI were also used as outcome indicator to compare. **a-c**, ROC curves based on PFS, MFS and RFI for integrated risk model, WHO classification, mDemicco model, and G-score, respectively in discovery SYSUCC cohort and the three validation cohorts (FAHSYSU and CHCAMS 1 and CHCAMS 2). Kaplan-Meier plots showing PFS for the patients stratified by integrated risk model (**d**) and WHO classification (**e**), MFS for mDemicco model (**f**), and RFI for G-score (**g**) in all primary non-CNS SFTs with NTM from four cohorts (n = 311), *p* values were calculated using two-sided log-rank test. **h-j**, Upset diagrams showing overlap of SFTs as low-risk, intermediate-risk and

high-risk group, stratified by integrated model, WHO classification, mDemicco model and G-score. PFS Progression Free Survival; MFS Metastasis Free Survival; RFI Recurrence Free Interval; WHO World Health Organization; HR Hazard Ratio; Source data are provided as a Source Data file.

Supplementary Table 1: Scoring of STAT6 immunohistochemical staining in the discovery cohort and three validation cohorts.

Cohorts	Score 3	Score 2	Score 1	Score 0
SYSUCC cohort	92 (70.23%)	28 (21.37%)	8 (6.11%)	3 (2.29%)
FAHSYSU cohort	85 (73.91%)	28 (24.35%)	2 (1.74%)	0 (0.00%)
CHCAMS cohort 1	83 (82.18%)	14 (13.86%)	3 (2.97%)	1 (0.99%)
CHCAMS cohort 2	49 (80.33%)	11 (18.03%)	1 (1.64%)	0 (0.00%)

Supplementary Table 2: Detection of *NAB2-STAT6* fusion gene by RT-PCR and sanger sequencing.

ID	Year of specimen	RNA concentration (ng/ul)	NAB2ex3-STAT6ex2	NAB2ex2-STAT6ex5	NAB2ex4-STAT6ex5	NAB2ex6-STAT6ex18	NAB2ex6-STAT6ex16	NAB2ex5-STAT6ex18	NAB2ex5-STAT6ex3	NAB2ex2-STAT6ex19	GAPDH
SYSUCC-SFT-5	2011	552.40	✓	×	×	×	×	×	×	×	✓
SYSUCC-SFT-12	2016	117.00	×	×	×	×	×	×	×	×	✓
SYSUCC-SFT-31	2013	920.50	✓	×	×	×	×	×	×	×	✓
SYSUCC-SFT-49	2013	214.50	×	×	×	×	×	×	×	×	✓
SYSUCC-SFT-58	2015	1082.00	✓	×	×	×	×	×	×	×	✓
SYSUCC-SFT-65	2014	245.80	✓	×	×	×	×	×	×	×	✓
SYSUCC-SFT-66	2018	89.20	×	×	×	×	×	×	×	×	✓
SYSUCC-SFT-71	2011	460.10	×	✓	×	×	×	×	×	×	✓
SYSUCC-SFT-76	2016	N/A	N/A	N/A	N/A	N/A	N/A	N/A	N/A	N/A	N/A
SYSUCC-SFT-84	2008	N/A	N/A	N/A	N/A	N/A	N/A	N/A	N/A	N/A	N/A
SYSUCC-SFT-89	2014	690.10	×	×	×	×	×	×	×	×	✓

× Not detected; ✓ Detected; N/A Not applied (failure of RNA extraction).

Supplementary Table 3: Univariate analysis for PFS in primary CNS SFTs with NTM from all four cohorts (n = 36).

Clinicopathologic feature	Number	Univariate analysis	
		HR (95%CI)	*P* value
Sex			0.383
Male	17 (47.22%)	1.67 (0.51-5.55)	
Female	19 (52.78%)	1.00	
Age at diagnosis (years)			0.987
≥ 55	11 (30.56%)	1.01 (0.30-3.46)	
< 55	25 (69.44%)	1.00	
Tumor size (cm)			0.413
D < 5	18 (50.00%)	1.00	
5 ≤ D < 10	17 (47.22%)	2.24 (0.68-7.34)	
10 ≤ D < 15	1 (2.78%)	0.34 (0.00-96.52)	
15 ≤ D	NA		
Cellularity			0.058
Low	14 (38.89%)	1.00	
Moderate	7 (19.44%)	3.98 (1.00-15.88)	
High	15 (41.67%)	0.96 (0.16-5.67)	
Necrosis			0.030
Absent	33 (91.67%)	1.00	
Present	3 (8.33%)	4.55 (0.28-75.17)	
Nuclear pleomorphism			0.350
Low	7 (19.44%)	1.00	
Moderate	22 (61.11%)	4.20 (1.06-16.60)	
High	7 (19.44%)	3.43 (0.33-35.85)	
Mitoses/10 HPF			0.013
0-4	17 (47.22%)	1.00	
≥ 5	19 (52.78%)	5.44 (1.66-17.82)	
2021 CNS WHO Grade			0.011
Grade1	17 (47.22%)	1.00	
Grade2	5 (13.89%)	2.50 (0.12-52.08)	
Grade3	14 (38.89%)	7.01 (1.93-25.42)	
Radiotherapy			0.091
No	26 (72.22%)	1.00	
Yes	10 (27.78%)	2.66 (0.56-12.49)	
Chemotherapy			0.080
No	35 (97.22%)	1.00	
Yes	1 (2.78%)	5.11 (0.07-379.81)	

HR Hazard Ratio; CI Confidence Interval; D Diameter; HPF High Power Field; CNS Central Nervous System; WHO World Health Organization; NA Not applied. *P* values were calculated using two-sided log-rank test.

Supplementary Table 4: Univariate analysis for PFS in relapsed non-CNS SFTs with NTM from all four cohorts (n = 31).

Clinicopathologic feature	Number	Univariate analysis	
		HR (95%CI)	*P* value
Sex			0.776
Male	18 (58.06%)	0.89 (0.38-2.07)	
Female	13 (41.94%)	1.00	
Age at diagnosis (years)			0.676
≥ 55	17 (54.84%)	0.85 (0.36-1.98)	
< 55	14 (45.16%)	1.00	
Tumor size (cm)			0.614
D < 5	15 (48.39%)	1.00	
5 ≤ D < 10	6 (19.35%)	0.72 (0.24-2.13)	
10 ≤ D < 15	5 (16.13%)	1.55 (0.42-5.81)	
15 ≤ D	5 (16.13%)	1.13 (0.36-3.52)	
Cellularity			0.198
Low	11 (35.48%)	1.00	
Moderate	14 (45.16%)	0.38 (0.12-1.21)	
High	6 (19.35%)	0.74 (0.24-2.30)	
Necrosis			0.098
Absent	29 (93.55%)	1.00	
Present	2 (6.45%)	3.09 (0.28-34.08)	
Nuclear pleomorphism			0.239
Low	5 (16.13%)	1.00	
Moderate	22 (70.97%)	2.87 (1.02-8.09)	
High	4 (12.90%)	4.29 (0.58-31.73)	
Specimen type			0.630
Recurrence	23 (74.19%)	0.81 (0.32-2.06)	
Metastasis	8 (25.81%)	1.00	
WHO classification			0.479
Locally aggressive, Benign	5 (16.13%)	1.00	
Rarely metastasizing, NOS	--	--	
Malignant	26 (83.87%)	1.53 (0.53-4.39)	
Mitoses / 10 HPF			0.172
< 4	8 (25.81%)	1.00	
≥ 4	23 (74.19%)	2.02 (0.82-4.98)	
Radiotherapy			0.268
No	27 (87.10%)	1.00	
Yes	4 (12.90%)	2.20 (0.28-17.05)	
Chemotherapy			0.639
No	25 (80.65%)	1.00	
Yes	6 (19.35%)	0.78 (0.29-2.13)	

HR Hazard Ratio; CI Confidence Interval; D Diameter; WHO World Health Organization; NOS Not Otherwise Specified; HPF High Power Field; -- Undefined, *P* values were calculated using two-sided log-rank test.

Supplementary Table 5: Detection of *IDH1* p.R132S mutation by sanger sequencing in seven SFTs in SYSUCC cohort.

ID	Variant
SYSUCC-SFT-3	p.R132S
SYSUCC-SFT-6	p.R132S
SYSUCC-SFT-49	p.R132S
SYSUCC-SFT-71	p.R132S
SYSUCC-SFT-101	p.R132S
SYSUCC-SFT-111	p.R132S
SYSUCC-SFT-117	p.R132S

Supplementary Table 6: Prediction of *MTOR* damaging mutation by Polyphen-2-HumDiv & SIFT & COSMIC.

ID	cHGVS & pHGVS	Polyphen-2-HumDiv	Score	SIFT	Score	COSMIC FATHMM prediction	Score	somatic
FAHSYSU-SFT-38	c.3940 G>A,p.A1314T	Probably damaging	0.993	Damaging	0.030	Pathogenic	0.980	yes
FAHSYSU-SFT-40	c.370 G>A,p.E124K	Probably damaging	0.968	Tolerated	0.301	-	-	-
FAHSYSU-SFT-45	c.3940 G>A,p.A1314T	Probably damaging	0.993	Damaging	0.030	Pathogenic	0.980	yes
	c.3853 A>T,p.R1285*	-	-	-	-	-	-	-
FAHSYSU-SFT-49	c.370 G>A,p.E124K	Probably damaging	0.968	Tolerated	0.301	-	-	-
FAHSYSU-SFT-69	c.370 G>A,p.E124K	Probably damaging	0.968	Tolerated	0.301	-	-	-
FAHSYSU-SFT-76	c.331 C>T,p.L111F	Probably damaging	1.000	Damaging	0.002	-	-	-
FAHSYSU-SFT-90	c.3823 G>A, p.V1275I	Possibly damaging	0.841	Tolerated	0.264	-	-	-
	c.3839 G>A, p.W1281*	-	-	-	-	-	-	-
	c.3935 C>T, p.P1312L	Probably damaging	1.000	Damaging	0.006	-	-	-
	c.3812C>A, p.A1271D	Probably damaging	0.998	Damaging	0.004	-	-	-
FAHSYSU-SFT-101	c.323 C>T,p.A108V	Possibly damaging	0.870	Damaging	0.022	-	-	-
FAHSYSU-SFT-102	c.5536 G>A,p.G1846S	Benign	0.128	Tolerated	0.271	-	-	-
	c.5555 A>G,p.E1852G	Probably damaging	0.991	Tolerated	0.087	-	-	-
FAHSYSU-SFT-114	c.5455 C>T,p.H1819Y	Possibly damaging	0.925	Tolerated	0.168	Pathogenic	0.990	yes
	c.5582 C>T,p.T1861I	Benign	0.006	Tolerated	0.253	-	-	-
CHCAMS-SFT-39	c.3912 G>A, p.W1304*	-	-	-	-	Pathogenic	0.990	yes
CHCAMS-SFT-40	c.3805 T>C, p.W1269R	Probably damaging	0.990	Damaging	0.000	-	-	-
CHCAMS-SFT-65	c.433 G>A, p.V145M	Probably damaging	0.995	Damaging	0.002	-	-	-
CHCAMS-SFT-90	c.3844 G>A, p.E1282K	Possibly damaging	0.956	Damaging	0.000	-	-	-
SYSUCC-SFT-14	c.2405T>C, p.I802T	Probably damaging	0.932	Damaging	0.001	-	-	-
SYSUCC-SFT-60	c.3811G>A, p.A1271T	Probably damaging	0.999	Tolerated	0.423	-	-	-
SYSUCC-SFT-98	c.376G>A, p.A126T	Possibly damaging	0.761	Damaging	0.018	-	-	-
SYSUCC-SFT-130	c.5506G>A, p.A1836T	Probably damaging	0.980	Tolerated	0.642	-	-	-
CHCAMS2-SFT-2	c.3820 A>T, p.R1274W	Possibly damaging	0.758	-	-	-	-	-
	c.3834 AT>TG, p.D1278V	Probably damaging	0.998	-	-	-	-	-
CHCAMS2-SFT-23	c.3844 G>A, p.E1282K	Possibly damaging	0.956	-	-	-	-	-
	c.3854 G>A, p.R1285K	Possibly damaging	0.841	-	-	-	-	-
CHCAMS2-SFT-58	c.2410 G>C, p.E804Q	Possibly damaging	0.808	-	-	-	-	-

Supplementary Table 7: Univariable analysis of the density of immune markers and Ki-67 with patient PFS in the SYSUCC cohort.

Immune microenviropment feature & Ki-67 (cells/mm²)	Number (%)	Univariate analysis	
		HR (95%CI)	*P* value
CD3+ cells density			0.215
≥ 140.00	61 (46.56%)	0.62 (0.29-1.30)	
< 140.00	70 (53.44%)	1.00	
CD8+ cells density			0.074
≥ 21.20	63 (48.09%)	2.01 (0.96-4.22)	
< 21.20	68 (51.91%)	1.00	
CD68+ cells density			0.003
≥ 80.60	43 (32.82%)	2.93 (1.32-6.52)	
< 80.60	88 (67.18%)	1.00	
CD163+ cells density			2.498E-06
≥ 929.30	38 (29.01%)	4.93 (1.99-12.18)	
< 929.30	93 (70.99%)	1.00	
FOXP3+ cells density			0.003
≥ 3.80	118 (90.08%)	0.26 (0.05-1.35)	
< 3.80	13 (9.92%)	1.00	
CD11c+ cells density			0.104
≥ 13.20	91 (69.47%)	2.18 (0.99-4.82)	
< 13.20	40 (30.53%)	1.00	
CD4+ cells density			0.016
≥ 495.10	58 (44.27%)	2.50 (1.18-5.30)	
< 495.10	73 (55.73%)	1.00	
Ki-67+ cells density			1.110E-16
≥ 454.70	18 (13.74%)	11.58 (2.94-45.51)	
< 454.70	113 (86.26%)	1.00	
HLA-DPB1+ cells density			0.007
≥ 4857.30	18 (13.74%)	2.94 (0.94-9.21)	
< 4857.30	113 (86.26%)	1.00	
PD-L1 expression			0.419
High expression in tumor cell	18 (13.74%)	1.32 (0.40-4.30)	
High expression in immune cell	14 (10.69%)	1.89 (0.56-6.42)	
Others	99 (75.57%)	1	
CD20+ cells density			0.010
≥ 16.5	113 (86.26%)	0.35 (0.12-1.09)	
< 16.5	18 (13.74%)	1.00	

HR Hazard Ratio; CI Confidence Interval; *P* values were calculated using two-sided log-rank test.

Supplementary Table 8: Cumulative survival analysis of the patients stratified by integrated risk model in discovery cohort and three validation cohorts of primary non-CNS SFTs with NTM.

Cohorts	Number (%)	3 years PFS	5 years PFS	10 years PFS
SYSUCC cohort, n = 101				
Low	63 (62.38%)	100%	100%	100%
Intermediate	31 (30.69%)	89%	67%	32%
High	7 (6.93%)	43%	NA[a]	NA
TFAHSYSU cohort, n = 71				
Low	23 (32.39%)	100%	100%	NA
Intermediate	30 (42.25%)	95%	80%	NA
High	18 (25.35%)	48%	24%	NA
CHCAOMS cohort 1, n = 84				
Low	44 (52.38%)	100%	100%	NA
Intermediate	31 (36.90%)	75%	NA	NA
High	9 (10.71%)	76%	NA	NA
CHCAOMS cohort 2, n = 55				
Low	28 (50.91%)	100%	100%	100%
Intermediate	19 (34.55%)	92%	83%	83%
High	8 (14.55%)	72%	18%	NA

PFS Progression Free Survival; NA Not Applied.

Supplementary Table 9: Cumulative survival analysis of the patients stratified by integrated risk model and WHO grade in discovery cohort (SYSUCC) and three validation cohorts of primary CNS SFTs with NTM.

Classification	Number (%)	3 years PFS	5 years PFS	10 years PFS
Integrated model				
Low	8 (22.22%)	100%	100%	NA
Intermediate	10 (27.78%)	100%	78%	NA
High	18 (50.00%)	38%	0%	0%
2021 CNS WHO Grade				
Grade1	17 (47.22%)	88%	66%	NA
Grade2	5 (13.89%)	71%	NA	NA
Grade3	14 (38.89%)	46%	10%	NA

PFS Progression Free Survival; CNS Central Nervous System; WHO World Health Organization; NA Not Applied.

Supplementary Table 10: C-index of integrated risk model, 2021 WHO grade in primary CNS SFTs with NTM from the four cohorts.

Classification	C-index (95%CI)	*P* value
Integrated model	0.901 (0.833-0.969)	4.88E-31
2021 WHO Grade	0.802 (0.709-0.895)	2.42E-10

CI Confidence Interval; WHO World Health Organization; *P* values were tested using the *z*-test. Source data are provided as a Source Data file.

Supplementary Table 11: C-index of integrated risk model, WHO classification in relapsed non-CNS SFTs with NTM from the four cohorts.

Classification	C-index (95%CI)	*P* value
Integrated model	0.550 (0.375-0.725)	0.576
WHO classification	0.520 (0.410-0.630)	0.721

CI Confidence Interval; WHO World Health Organization; *P* values were tested using the *z*-test. Source data are provided as a Source Data file.

Supplementary Table 12: C-index of integrated risk model, WHO classification, mDemicco model, and G-score of PFS, MFS, and RFI in discovery SYSUCC cohort and the three validation cohorts (FAHSYSU and CHCAMS 1 and CHCAMS 2).

Classification	C-index (95%CI)-PFS	*P* value	C-index (95%CI)-MFS	*P* value	C-index (95%CI)-RFI	*P* value
Integrated model	0.890 (0.848-0.932)	4.90E-75	0.907 (0.834-0.980)	2.73E-28	0.882 (0.834-0.930)	1.80E-54
WHO classification	0.773 (0.693-0.853)	3.09E-11	0.820 (0.703-0.937)	7.71E-08	0.750 (0.655-0.845)	2.27E-07
mDemicco model	0.809 (0.736-0.883)	1.43E-16	0.767 (0.607-0.927)	1.02E-03	0.782 (0.694-0.870)	3.45E-10
G-score	0.784 (0.691-0.877)	2.56E-09	0.734 (0.573-0.895)	4.40E-03	0.743 (0.636-0.850)	7.52E-06

CI Confidence Interval; PFS Progression Free Survival; MFS Metastasis Free Survival; RFI Recurrence Free Interval; WHO World Health Organization; *P* values were tested using the *z*-test. Source data are provided as a Source Data file.

Supplementary Table 13: Cumulative survival of the patients stratified by integrated risk model, WHO classification, mDemicco model, and G-score for all primary non-CNS SFTs with NTM (n = 311).

Classification	Number (%)	3 yeras-PFS	5 years-PFS	10 yeras-PFS	3 yeras-MFS	5 years-MFS	10 yeras-MFS	3 yeras-RFI	5 years-RFI	10 yeras-RFI
WHO classification										
Locally aggressive, Benign	243 (78.14%)	96%	93%	91%	100%	98%	98%	97%	93%	92%
Rarely metastasizing, NOS	15 (4.82%)	92%	92%	NA	92%	92%	NA	92%	92%	NA
Malignant	53 (17.04%)	63%	40%	26%	88%	61%	61%	73%	46%	30%
Integrated model										
Low	158 (50.80%)	100%	100%	100%	100%	100%	100%	100%	100%	100%
Intermediate	111 (35.69%)	87%	73%	57%	98%	87%	87%	90%	75%	58%
High	42 (13.50%)	59%	21%	NA	85%	42%	NA	71%	25%	NA
mDemicco										
Low	229 (73.63%)	96%	93%	91%	99%	99%	99%	97%	94%	92%
Intermediate	62 (19.94%)	83%	71%	71%	92%	79%	79%	87%	75%	75%
High	20 (6.43%)	56%	25%	NA	93%	54%	NA	68%	31%	NA
G-score										
Low	117 (37.62%)	96%	90%	90%	99%	99%	99%	96%	90%	90%
Intermediate	142 (45.66%)	94%	88%	88%	97%	93%	93%	94%	88%	88%
High	52 (16.72%)	67%	50%	27%	92%	69%	69%	80%	60%	32%

PFS Progression Free Survival; MFS Metastasis Free Survival; RFI Recurrence Free Interval; WHO World Health Or ganization; NOS Not Otherwise Specified; NA Not Applied.

Supplementary Table 14: The detailed information of the antibody used.

Target Protein	Host	Vendor	Catalog	Clone	Function	Dilution	RRID
STAT6	Rabbit	MXB Biotechnologies	RMA-0845	EP325	Tumor cells	Ready to use	AB_778113
Ki-67	Rabbit	MXB Biotechnologies	RMA-0731	MXR002	Proliferation	Ready to use	--
CD68	Mouse	MXB Biotechnologies	Kit-0026	KP1	Macrophage	Ready to use	--
CD163	Mouse	MXB Biotechnologies	MAB-0206	10D6	Macrophage	Ready to use	--
HLA-DPB1	Rabbit	Abcam	ab157210	EPR11226	T cells,Tumor cells	1:3000	AB_2827533
PD-L1	Rabbit	Cell signaling technology	Cat# 13684S	E1L3N	Tumor cells, Macrophage	1:300	AB_2687655
CD3	Rabbit	MXB Biotechnologies	Kit-0003	SP7	T cell	Ready to use	--
CD4	Rabbit	MXB Biotechnologies	RMA-0620	SP35	T cell	Ready to use	--
CD8	Rabbit	MXB Biotechnologies	RMA-0514	SP16	T cell	Ready to use	--
FOXP3	Mouse	Biolegend	320102	206D	Treg cell	1:400	AB_430881
CD11c	Rabbit	Abcam	ab52632	EP1347Y	Dendritic cell	1:300	AB_2129793
CD20	Rabbit	MXB Biotechnologies	Kit-0001	L26	B cell	Ready to use	--
CD68	Rabbit	Cell signaling technology	79594S	D4B9C	Macrophage	1:100	AB_2799935
CD163	Rabbit	Abcam	ab218294	EPR14643-36	Macrophage	1:100	AB_2889155
CD206	Mouse	Santa Cruz Biotechnology	sc-376108 AF488 D-1	D-1	Macrophage	1:100	AB_10987732
STAT6	Rabbit	Abcam	ab207014	YE361	Tumor cells	1:100	AB_2889256
PD-L1	Rabbit	Abcam	ab267563	SP142	Tumor cells, Macrophage	1:100	AB_2827816
PD1	Rabbit	Abcam	ab201825	EPR4877(2)	T cells	1:100	AB_2728811
CD8a	Mouse	eBioscience	50-0008-80	AMC908	T cells	1:100	AB_2574148
CD4	Mouse	eBioscience	41-2444-80	N1UG0	T cells	1:100	AB_2573601
Secondary Antibody, Alexa Fluor 488	Chicken anti-Goat	ThermoFisher	A-21467	--	--	1:500	--
Secondary Antibody, Alexa Fluor 555	Goat anti-Rabbit	ThermoFisher	A-21428	--	--	1:500	--
Secondary Antibody Alexa Fluor 647	Chicken anti-Mouse	ThermoFisher	A-21463	--	--	1:500	--
Secondary Antibody	anti-Mouse/Rabbit	MXB Biotechnologies	KIT-5020	--	--	Ready to use	--

Supplementary Table 15: PCR Primer sequences for *MTOR* and *IDH1*.

Gene	Primer sequence	Tm
MTOR		
Fm-1	5'- CATACGCCCCATCATCACC	60℃
Rm-1	5'- ATTCCACGTACTCAGCGGTA	
Fm-2	5'- CATAACTTCTTTCTCTAGGCAT	57℃
Rm-2	5'- CCGCAATAAATATGTGAGACC	
Fm-3	5'- ACTCTAGGGGAATTTTATCGT	55℃
Rm-3	5'- GCCCTGACACACTATACCTG	
Fm-4	5'- TTGCCTTTCTCCCCAACCAG	59°C
Rm-4	5'- TGGCACTTCAGATACAGC	
IDH1		
Forward	5'- TGTGTTGAGATGGACGCCTATTTG	56℃
Reverse	5'- TGCCACCAACGACCAAGTCA	

Tm Melting Temperature; F Forward; R Reverse.

Supplementary Table 16: Sequence of primers used for *NAB2-STAT6* fusion.

NAB2-STAT6	Primer sequence	Fusion variants	Size(s) of PCR product(s) obtained	Tm (°C)
NAB2 ex3 forward	5'- GCCCGAGAGAGCACCTACTT	4-2	173bp	58
STAT6 ex2 reverse	5'-ACATAGAGCCGCTGCACTTT			
NAB2 ex4 forward	5'-GCTTCACCCTGAAGAACTGG	4-4	202bp	58
STAT6 ex5 reverse	5'-CCGCAAGCCTGTCTTAAACT			
NAB2 ex6 forward	5'-ACATCCTGCAGCAGACACTG	6-17; 6-16	199bp; 346bp	58
STAT6 ex18 reverse	5'-TCTGGGGTAGGAAGTGGTTG			
NAB2 ex6 forward	5'-AGCAGACACTGATGGACGAG	6-16	219bp	58
STAT6 ex16 reverse	5'-TGGGCTTCTTGGGATAGAGA			
NAB2 ex5 forward	5'-GTCACCCTGAAATCCAGCAG	6-18	403bp	58
STAT6 ex18 reverse	5'-CATGCTCATGGAGGAATCAG			
NAB2 ex2 forward	5'-CATCTATGGCCGTTTCGACT	2-5	124bp	58
STAT6 ex5 reverse	5'-CCGCAAGCCTGTCTTAAACT			
NAB2 ex5 forward	5'-GTCTGGGGAGAGTCTGGATG	5-3; 6-3	106bp; 298bp	58
STAT6 ex3 reverse	5'-GGTGCTGGACAGTGTCTGAA			
NAB2 ex2 forward	5'-CATCTATGGCCGTTTCGACT	3-19	228bp	58
STAT6 ex19 reverse	5'-GGGATGGAGTGAGAGTGTGG			
GAPDH forward	5'-GGAGCGAGATCCCTCCAAAAT		197bp	58
GAPDH reverse	5'-GGCTGTTGTCATACTTCTCATGG			

PCR Polymerase Chain Reaction; Tm Melting Temperature.
