# Supplementary material for: Comprehensive analysis reveals potential therapeutic targets and an integrated risk stratification model for solitary fibrous tumors
Source: Nat Commun. 2023 Nov 18;14:7479. doi: 10.1038/s41467-023-43249-4 (PMC10657378; doi:10.1038/s41467-023-43249-4)
Supplement: Supplementary file 3 — Description of Additional Supplementary Files [file 41467_2023_43249_MOESM3_ESM.pdf]

Description of Additional Supplementary Files

File Name: Supplementary Data 1
Description: Detailed information for each SFT patient in SYSUCC cohort and three validation cohorts (FAHSYSU cohort and CHCAMS cohort 1 and CHCAMS cohort 2) (n = 408).

File Name: Supplementary Data 2
Description: Detailed information on altered genes detected by NGS in 131 SFTs in SYSUCC cohort.

File Name: Supplementary Data 3
Description: Detection of *MTOR* mutation by sanger sequencing in FAHSYSU cohort and CHCAMS cohort 1 and CHCAMS cohort 2.
