# Supplementary material for: Comprehensive analysis reveals potential therapeutic targets and an integrated risk stratification model for solitary fibrous tumors
Source: Nat Commun. 2023 Nov 18;14:7479. doi: 10.1038/s41467-023-43249-4 (PMC10657378; doi:10.1038/s41467-023-43249-4)
Supplement: Supplementary file 7 — Reporting Summary [file 41467_2023_43249_MOESM7_ESM.pdf]

nature portfolio

Corresponding author(s): Qin Li
Yan-Fen Feng
Ziming Du

Last updated by author(s): Oct 16, 2023

Reporting Summary

Nature Portfolio wishes to improve the reproducibility of the work that we publish. This form provides structure for consistency and transparency in reporting. For further information on Nature Portfolio policies, see our Editorial Policies and the Editorial Policy Checklist.

Statistics

For all statistical analyses, confirm that the following items are present in the figure legend, table legend, main text, or Methods section.

n/a	Confirmed	
☐	☒	The exact sample size (*n*) for each experimental group/condition, given as a discrete number and unit of measurement
☐	☒	A statement on whether measurements were taken from distinct samples or whether the same sample was measured repeatedly
☐	☒	The statistical test(s) used AND whether they are one- or two-sided<br>*Only common tests should be described solely by name; describe more complex techniques in the Methods section.*
☐	☒	A description of all covariates tested
☐	☒	A description of any assumptions or corrections, such as tests of normality and adjustment for multiple comparisons
☐	☒	A full description of the statistical parameters including central tendency (e.g. means) or other basic estimates (e.g. regression coefficient) AND variation (e.g. standard deviation) or associated estimates of uncertainty (e.g. confidence intervals)
☐	☒	For null hypothesis testing, the test statistic (e.g. *F*, *t*, *r*) with confidence intervals, effect sizes, degrees of freedom and *P* value noted<br>*Give P values as exact values whenever suitable.*
☒	☐	For Bayesian analysis, information on the choice of priors and Markov chain Monte Carlo settings
☒	☐	For hierarchical and complex designs, identification of the appropriate level for tests and full reporting of outcomes
☒	☐	Estimates of effect sizes (e.g. Cohen's *d*, Pearson's *r*), indicating how they were calculated

Our web collection on statistics for biologists contains articles on many of the points above.

Software and code

Policy information about availability of computer code

Data collection	Targeted next generation sequencing (NGS) was performed using the GenePlus 2000 Sequencing platform (Geneplus-Beijing institute, China) .<br>Sanger sequencing was performed by ABI 3500XL Genetic Analyzer (Applied Biosystems, USA).<br>The immunohistochemistry slides were scanned using a digital pathology scanner (Axio Scan.Z1, Germany) to acquire digital images that were quantitatively scored using the HALO 2.3 digital pathology system (Indica Labs).<br>Images of multiplex immunofluorescence staining were acquired using a confocal microscope (LSM 980, Carl ZEISS) with a 20x0.80 objective.
Data analysis	Commercial software: SPSS statistical software version 17.0, Graphpad prism 8.0, Microsoft Office 365(Excel, Word).<br>Open source software: R software version 4.1.3 including some pakage such as ComplexHeatmap v2.14.0, survival v3.4.0, ggpubr v0.6.0, ggplot2 v3.4.1, ggpubr v0.6.0, randomForestSRC v3.2.0, rms v6.5.0, Hmisc v4.8.0, pROC v1.18.0, ggalluvial v0.12.4, UpSetR v1.4.0 were used for statistical analyses or graphic presentation, BWA18 (version 0.7.12-r1039), MuTect19 (version 1.1.4) , ANNOVAR20, CONTRA21, PolyPhen2/SIFT (ensdb v73) and Cosmic (V80).<br>The custom code used to analyze the images of multiplex immunofluorescence staining was published previously (Du Z, et al. Nat Protoc, 2019 Oct;14(10):2900-2930. doi: 10.1038/s41596-019-0206-y), and is available on GitHub (https://github.com/sorgerlab/cycif).

For manuscripts utilizing custom algorithms or software that are central to the research but not yet described in published literature, software must be made available to editors and reviewers. We strongly encourage code deposition in a community repository (e.g. GitHub). See the Nature Portfolio guidelines for submitting code & software for further information.

Data

Policy information about availability of data

All manuscripts must include a data availability statement. This statement should provide the following information, where applicable:

- Accession codes, unique identifiers, or web links for publicly available datasets
- A description of any restrictions on data availability
- For clinical datasets or third party data, please ensure that the statement adheres to our policy

Source data are provided with this paper. Data used in the preparation of this manuscript are available within the Article, Supplementary Information, Supplementary Data and Source Data file. There are no restrictions on data access.

Raw next generation sequencing data has been deposited in Genome Sequence Archive of National Genomics Data Center with bioProject accession: PRJCA015954, according to Guidance of the Ministry of Science and Technology (MOST) for the Review and Approval of Human Genetic Resources https://bigd.big.ac.cn/gsa-human/browse/HRA004309.

Raw imaging data files from immunohistochemistry and multiplex immunofluorescence staining are substantial and can be made available from the corresponding authors on request. Further information and requests for resources and reagents should be directed to and will be promptly fulfilled by the corresponding authors.

Research involving human participants, their data, or biological material

Policy information about studies with human participants or human data. See also policy information about sex, gender (identity/presentation), and sexual orientation and race, ethnicity and racism.

Reporting on sex and gender	A total of 408 SFT patients were recruited in this study, in which 195 male and 213 female SFT patients were included. Our research involves human research participants, and both female and male patients were included in our study. Sex was considered in our study design and the sex of the participants were determined by self-report and also biology characteristics. Additionally, we conducted a priori sex-and gender-based analyses and have reported the results.
Reporting on race, ethnicity, or other socially relevant groupings	Race, ethnicity, or other socially relevant groupings were not involved in our study.
Population characteristics	Total four cohorts consisted of 195 male and 213 female SFT patients, and had a median age of 51 (9 - 87) years old.
Recruitment	We collected formalin-fixed paraffin-embedded (FFPE) tumor tissue specimens from 131 histologically proven SFT cases at the Sun yat-sen University Cancer Center (SYSUCC) between 2008 and 2020, forming the discovery cohort (SYSUCC cohort). Additionally, we recruited 277 FFPE tumor tissue specimens from two other hospitals to establish three independent validation cohorts. These validation cohorts included 115 cases from the First Affiliated Hospital of Sun yat-sen University between 2013 and 2021 (FAHSYSU cohort), 101 cases from the Cancer Hospital Chinese Academy of Medical Sciences between 2017 and 2021 (CHCAMS cohort 1), and 61 cases from CHCAMS between 2013 and 2016 (CHCAMS cohort 2). The inclusion criteria were as follows: (i) All tumors were histologically verified as SFT. (ii) None of the tumor samples received chemotherapy , radiotherapy, or targeted therapy prior to surgery. (iii) All the patients had complete clinicopathological data.
Ethics oversight	This study was approved by the Regional Ethics Committees at all participating institutions (Ethics Committee of Sun Yat-sen University Cancer Center : B2021-421-01; Ethics Committee of The First Affiliated Hospital, Sun Yat-sen University: [2022]409; Ethics Committee of National Cancer Center/Cancer Hospital, Chinese Academy of Medical Sciences and Peking Union Medical College: 22/024-3225). The requirement for informed consent was waived, and no compensation was provided to the participants in this study.

Note that full information on the approval of the study protocol must also be provided in the manuscript.

Field-specific reporting

Please select the one below that is the best fit for your research. If you are not sure, read the appropriate sections before making your selection.

☒ Life sciences ☐ Behavioural & social sciences ☐ Ecological, evolutionary & environmental sciences

For a reference copy of the document with all sections, see nature.com/documents/nr-reporting-summary-flat.pdf

Life sciences study design

All studies must disclose on these points even when the disclosure is negative.

Sample size	We collected formalin-fixed paraffin-embedded (FFPE) tumor tissue specimens from 131 histologically proven SFT cases at the Sun yat-sen University Cancer Center (SYSUCC) between 2008 and 2020, forming the discovery cohort (SYSUCC cohort). Additionally, we recruited 277 FFPE tumor tissue specimens from two other hospitals to establish three independent validation cohorts. These validation cohorts included

	115 cases from the First Affiliated Hospital of Sun yat-sen University between 2013 and 2021 (FAHSYSU cohort), 101 cases from the Cancer Hospital Chinese Academy of Medical Sciences between 2017 and 2021 (CHCAMS cohort 1), and 61 cases from CHCAMS between 2013 and 2016 (CHCAMS cohort 2) . Targeted next-generation sequencing (NGS) was performed in the discovery cohort (131 cases), while the immunohistochemistry was performed in all three cohorts (408 cases). We used all available samples for analysis, and the sample sizes were not pre-determined through stattitical criteria, but a large number of specimens robustly exceeding the sample sizes from the literatures about SFT studies.
Data exclusions	We used the following inclusion criteria to exclude the SFT patients: (i) All tumors were histologically verified as SFT. (ii) None of the tumor samples received chemotherapy, radiotherapy, or targeted therapy prior to surgery. (iii) All the patients had complete clinicopathological data. For risk modeling, we only used primary non-CNS SFTs with tumor margin negative in SYSUCC cohort (n = 101) for the integrated risk model generation, which was then validated in three cohorts with the same criteria. Additionally, the integrated risk model was also tested in both primary CNS SFTs with tumor margin negative and the relapsed (recurrent and metastatic) non-CNS SFTs with tumor margin negative.
Replication	The NGS, IHC, and multiplexed immunofluorescence staining were conducted in a single experimental run. However, the experimental analyses, along with their respective analysis pipelines, including NGS data analysis, IHC image analysis, multiplexed immunofluorescence image analysis, and risk modeling, were technically reproducible. The main findings were cross-validated across different cohorts.
Randomization	Randomization was not applicable in this study as it involves a retrospective analysis of large SFT specimen cohorts, and there was no prospective patient enrollment. The SYSUCC internal cohort (2008-2020) served as the discovery cohort, whereas the external validation cohorts comprised data from FAHSYSU (2013-2021), CHCAMS 1 (2017-2021), and CHCAMS 2 (2013-2016).
Blinding	The investigators was blind to clinical information while performing experiments and analysis.

Reporting for specific materials, systems and methods

We require information from authors about some types of materials, experimental systems and methods used in many studies. Here, indicate whether each material, system or method listed is relevant to your study. If you are not sure if a list item applies to your research, read the appropriate section before selecting a response.

Materials & experimental systems

n/a	Involved in the study
☐	☒ Antibodies
☒	☐ Eukaryotic cell lines
☒	☐ Palaeontology and archaeology
☒	☐ Animals and other organisms
☒	☐ Clinical data
☒	☐ Dual use research of concern
☒	☐ Plants

Methods

n/a	Involved in the study
☒	☐ ChIP-seq
☒	☐ Flow cytometry
☒	☐ MRI-based neuroimaging

Antibodies

Antibodies used

STAT6 (Ready to use, EP325, Cat# RMA-0845, MXB Biotechnologies),
Ki-67 (Ready to use, MXR002, Cat# RMA-0731, MXB Biotechnologies),
CD68 (Ready to use, KP1, Cat# Kit-0026, MXB Biotechnologies),
CD163 (Ready to use, 10D6, Cat# MAB-0206, MXB Biotechnologies),
HLA-DPB1 (1:3000 dilution, EPR11226, Cat# ab157210, Abcam),
PD-L1 (1:500 dilution, E1L3N, Cat# 13684S, Cell signaling technology),
CD3 (Ready to use, SP7, Cat# Kit-0003, MXB Biotechnologies),
CD4 (Ready to use, SP35, Cat# RMA-0620, MXB Biotechnologies),
CD8 (Ready to use, SP16, Cat# RMA-0514, MXB Biotechnologies),
FOXP3 (1:400 dilution, 206D, Cat# 320102, Biolegend),
CD11c (1:300 dilution, EP1347Y, Cat# ab52632, Abcam),
CD20 (Ready to use, L26, Kit-0001, MXB Biotechnologies),
MaxVision TM HRP-Polymer anti-Mouse/Rabbit IHC Kit, Secondary Antibody (Ready to use, Cat# KIT-5020, MXB Biotechnologies).
CD68 (1:100 dilution, D4B9C, Cat# 79594S, Cell Signaling Technology),
CD163 (1:100 dilution, EPR14643-36, Cat# ab218294, Abcam),
CD206 (1:100 dilution, D-1, Cat# sc-376108, Santa Cruz Biotechnology),
STAT6 (1:100 dilution, YE361, Cat# ab207014, Abcam),
PD-L1 (1:100 dilution, SP142, Cat# ab267563, Abcam),
PD1 (1:100 dilution, EPR4877(2), Cat# ab201825, Abcam),
CD4 (1:100 dilution, N1UG0, Cat# 41-2444-80, eBioscience),
CD8a (1:100 dilution, AMC908, Cat#50-0008-80, eBioscience)
Chicken anti-Goat IgG (H+L) Cross-Adsorbed Secondary Antibody, Alexa Fluor 488 (1:500 dilution, Cat# A-21467, ThermoFisher),
Goat anti-Rabbit IgG (H+L) Cross-Adsorbed Secondary Antibody, Alexa Fluor 555 (1:500 dilution, Cat# A-21428, ThermoFisher),
Chicken anti-Mouse IgG (H+L) Cross-Adsorbed Secondary Antibody, Alexa Fluor 647 (1:500 dilution, Cat# A-21463, ThermoFisher).

Validation

Antibody validation was performed by comparing signal between positive and negative tissue samples, the sub-cellular localization (e.g., membranous, cytoplasmic, nuclear), and multiple marker colocalization or lack of co-localization with markers known not to co-localize.
STAT6 (1:100 dilution, YE361, Cat# ab207014, Abcam),
https://www.abcam.cn/products/primary-antibodies/alexa-fluor-555-stat6-antibody-ye361-ab207014.html
-Validated for immunofluorescence in human HeLa cells by the manufacturer, immunofluorescence in paraffin-embedded human tissues with appropriate positive and negative controls in our lab.
CD163 (Ready to use, 10D6, Cat# MAB-0206, MXB Biotechnologies),
-With appropriate positive and negative controls, validated for immunohistochemistry (IHC) in paraffin-embedded human tissues by our lab.
CD3 (Ready to use, SP7, Cat# Kit-0003, MXB Biotechnologies),
-With appropriate positive and negative controls, validated for immunohistochemistry (IHC) in paraffin-embedded human tissues by our lab.

Multiple antibodies used in this study have been previously published, including:
FOXP3 (1:400 dilution, 206D, Cat# 320102, Biolegend),
CD11c (1:300 dilution, EP1347Y, Cat# ab52632, Abeam).
CD68 (1:100 dilution, D4B9C, Cat# 79594S, Cell Signaling Technology),
CD163 (1:100 dilution, EPR14643-36, Cat# ab218293, Abeam),
CD206 (1:100 dilution, D-1, Cat# sc-376108, Santa Cruz Biotechnology),
PD-L1 (1:100 dilution, SP142, Cat# ab267563, Abeam),
Chicken anti-Goat IgG (H+L) Cross-Adsorbed Secondary Antibody, Alexa Fluor 488 (1:500 dilution, Cat# A-21467, ThermoFisher),
Goat anti-Rabbit IgG (H+L) Cross-Adsorbed Secondary Antibody, Alexa Fluor 555 (1:500 dilution, Cat# A-21428, ThermoFisher),
Chicken anti-Mouse IgG (H+L) Cross-Adsorbed Secondary Antibody, Alexa Fluor 647 (1:500 dilution, Cat# A-21463, ThermoFisher).
Du z, et al. Nat Protec. 2019 Oct;14(10):2900-2930. doi: 10.1038/541596-019--0206-y.
Nirmal et al. Cancer Discov. 2022 Jun 2;12(6):1518-1541. doi: 10.1158/2159-8290.CD-21-1357

Antibodies used in this study were validated by the manufacturer, including:
STAT6 (Ready to use, EP325, Cat# RMA-0845, MXB Biotechnologies),
http://www.maxim.com.cn/sitecn/dklkthdklkt/7512.html
-Validated for immunohistochemistry (IHC) in paraffin-embedded human tissues by the manufacturer
Ki-67 (Ready to use, MXR002, Cat# RMA-0731, MXB Biotechnologies),
http://www.maxim.com.cn/sitecn/dklkthdklkt/7211.html
-Validated for immunohistochemistry (IHC) in paraffin-embedded human tissues by the manufacturer
CD68 (Ready to use, KP1, Cat# Kit-0026, MXB Biotechnologies),
http://www.maxim.com.cn/sitecn/dklkthdklkt/7076.html
-Validated for immunohistochemistry (IHC) in paraffin-embedded human tissues by the manufacturer
HLA-DPB1 (1:3000 dilution, EPR11226, Cat# ab157210, Abcam),
https://www.abcam.cn/products/primary-antibodies/hla-dpb1-antibody-epr11226-ab157210.html
-Validated for immunohistochemistry (IHC)/immunofluorescence in paraffin-embedded human tissues by the manufacturer
PD-L1 (1:500 dilution, E1L3N, Cat# 13684S, Cell signaling technology),
https://www.cellsignal.com/products/primary-antibodies/pd-l1-e1l3n-xp-rabbit-mab/13684?site-search-type=Products&N=4294956287&Ntt=13684s&fromPage=plp&_requestid=7569463
-Validated for immunohistochemistry (IHC) in paraffin-embedded human tissues by the manufacturer
CD4 (Ready to use, SP35, Cat# RMA-0620, MXB Biotechnologies),
http://www.maxim.com.cn/sitecn/dklkthdklkt/7007.html
-Validated for immunohistochemistry (IHC) in paraffin-embedded human tissues by the manufacturer
CD8 (Ready to use, SP16, Cat# RMA-0514, MXB Biotechnologies),
http://www.maxim.com.cn/sitecn/dklkthdklkt/7013.html
-Validated for immunohistochemistry (IHC) in paraffin-embedded human tissues by the manufacturer
FOXP3 (1:400 dilution, 206D, Cat# 320102, Biolegend),
https://www.biolegend.com/en-us/products/purified-anti-human-foxp3-antibody-2897
-Validated for immunohistochemistry (IHC) in paraffin-embedded human tissues by the manufacturer/ in the literature
CD11c (1:300 dilution, EP1347Y, Cat# ab52632, Abcam),
https://www.abcam.cn/products/primary-antibodies/cd11c-antibody-ep1347y-c-terminal-ab52632.html
-Validated for immunohistochemistry (IHC) in paraffin-embedded human tissues by the manufacturer
CD20 (Ready to use, L26, Kit-0001, MXB Biotechnologies),
http://www.maxim.com.cn/sitecn/dklkthdklkt/7028.html
-Validated for immunohistochemistry (IHC) in paraffin-embedded human tissues by the manufacturer
MaxVision TM HRP-Polymer anti-Mouse/Rabbit IHC Kit, Secondary Antibody (Ready to use, Cat# KIT-5020, MXB Biotechnologies).
http://www.maxim.com.cn/sitecn/myzhjcxthsjh/1056.html
-Validated for immunohistochemistry (IHC) in paraffin-embedded human tissues by the manufacturer
PD1 (1:100 dilution, EPR4877(2), Cat# ab201825, Abcam),

https://www.abcam.cn/products/primary-antibodies/alexa-fluor-647-pd1-antibody-epr48772-ab201825.html
-Validated for immunohistochemistry (IHC)/immunofluorescence in paraffin-embedded human tissues by the manufacturer
CD4 (1:100 dilution, N1UG0, Cat# 41-2444-80, eBioscience),
https://www.thermofisher.cn/cn/zh/antibody/product/CD4-Antibody-clone-N1UG0-Monoclonal/41-2444-80
-Validated for immunohistochemistry (IHC)/immunofluorescence in paraffin-embedded human tissues by the manufacturer
CD8a (1:100 dilution, AMC908, Cat#50-0008-80, eBioscience)
https://www.thermofisher.cn/cn/zh/antibody/product/CD8a-Antibody-clone-AMC908-Monoclonal/50-0008-80
-Validated for immunohistochemistry (IHC)/immunofluorescence in paraffin-embedded human tissues by the manufacturer
Chicken anti-Goat IgG (H+L) Cross-Adsorbed Secondary Antibody, Alexa Fluor 488 (1:500 dilution, Cat# A-21467, ThermoFisher),
https://www.thermofisher.cn/cn/zh/antibody/product/Chicken-anti-Goat-IgG-H-L-Cross-Adsorbed-Secondary-Antibody-Polyclonal/A-21467
-Validated for immunofluorescence by the manufacturer
Goat anti-Rabbit IgG (H+L) Cross-Adsorbed Secondary Antibody, Alexa Fluor 555 (1:500 dilution, Cat# A-21428, ThermoFisher),
https://www.thermofisher.cn/cn/zh/antibody/product/Goat-anti-Rabbit-IgG-H-L-Cross-Adsorbed-Secondary-Antibody-Polyclonal/A-21428
-Validated for immunofluorescence by the manufacturer
Chicken anti-Mouse IgG (H+L) Cross-Adsorbed Secondary Antibody, Alexa Fluor 647 (1:500 dilution, Cat# A-21463, ThermoFisher),
https://www.thermofisher.cn/cn/zh/antibody/product/Chicken-anti-Mouse-IgG-H-L-Cross-Adsorbed-Secondary-Antibody-Polyclonal/A-21463
-Validated for immunofluorescence by the manufacturer.

Plants

Seed stocks

Not avaliable

Novel plant genotypes

Not avaliable

Authentication

Not avaliable
